# Electrical Stimulation in the Claustrum Area Induces a Deepening of Isoflurane Anesthesia in Rat

**DOI:** 10.3390/brainsci9110304

**Published:** 2019-11-01

**Authors:** Bogdan Pavel, Fabien Menardy, Diana Rotaru, Alexandru Catalin Paslaru, Camelia Acatrinei, Leon Zagrean, Daniela Popa, Ana-Maria Zagrean

**Affiliations:** 1 Division of Physiology and Neuroscience, Carol Davila University of Medicine and Pharmacy, 050474 Bucharest, Romania; bogdan.pavel@umfcd.ro (B.P.); catalin.paslaru@umfcd.ro (A.C.P.); camelia.acatrinei@yahoo.com (C.A.); leon.zagrean@umfcd.ro (L.Z.); 2 Institut de Biologie de l’Ecole Normale Supérieure (IBENS), Ecole Normale Supérieure, CNRS, INSERM, PSL Research University, 75005 Paris, France; fabien.menardy@biologie.ens.fr; 3 Department of Neuroimaging, Centre for Neuroimaging Sciences, Institute of Psychiatry, Psychology and Neuroscience, King’s College London, London SE58AF, UK; diana.rotaru@kcl.ac.uk

**Keywords:** claustrum, electrical stimulation, anesthesia, isoflurane, burst suppression

## Abstract

The role of the claustrum in consciousness and vigilance states was proposed more than two decades ago; however, its role in anesthesia is not yet understood, and this requires more investigation. The aim of our study was to assess the impact of claustrum electrical stimulation during isoflurane anesthesia in adult rats. The claustrum in the left hemisphere was electrically stimulated using a bipolar tungsten electrode inserted stereotaxically. In order to monitor the anesthetic depth, the electrocorticogram (ECoG) was recorded before, during, and after claustrum stimulation using frontal and parietal epidural electrodes placed over the left hemisphere. After reaching stabilized slow-wave isoflurane anesthesia, twenty stimuli, each of one second duration with ten seconds interstimulus duration, were applied. ECoG analysis has shown that, after a delay from the beginning of stimulation, the slow-wave ECoG signal changed to a transient burst suppression (BS) pattern. Our results show that electrical stimulation of the claustrum area during slow-wave isoflurane anesthesia induces a transitory increase in anesthetic depth, documented by the appearance of a BS ECoG pattern, and suggests a potential role of claustrum in anesthesia.

## 1. Introduction

The claustrum is a subcortical structure of the brain, first described by Thomas Willis in 1672 [1]. Recent studies have revealed that this structure interacts with almost all other structures of the brain: motor cortex, somatosensory cortex, prefrontal cortex, cingulate cortex, auditory and visual cortex, hippocampus, amygdala, and caudate nucleus [2]. The established connections are mainly ipsilateral, with sparse contralateral connections [3].

Based on anatomy and physiology studies, Francis Crick suggested the involvement of the claustrum in maintaining consciousness [4]. In 2014, Koubeissi et al. noticed that left claustrum stimulation in humans resulted in the abolition of consciousness for the whole duration of stimulation [5]. On the other hand, in a recent study, Bickel and Parvizi reported that bilateral stimulation of the claustrum in humans failed to induce a loss of consciousness [6]. One major aspect to be considered when comparing the two studies is that the protocol used by Bickel and Parvizi [6] was fairly different from the one presented by Koubeissi et al. [5]. Additionally, in 2015 Chau et al. studied a group of veterans who suffered from traumatic brain injuries, which also included lesions in the claustrum. They concluded that the claustrum was involved in regaining consciousness, rather than maintaining it [7].

Anesthesia, characterized as a loss of consciousness, is administered daily to millions of patients but remains a continuous mystery for researchers and anesthetists. More than 170 years after the beginning of modern anesthesia, it is still unknown where and how anesthetics work. Classically, anesthetics are thought to work on the spinal cord, the brainstem, the thalamus, and the cerebral cortex [8,9]. Although the claustrum is closely interconnected with all cortical and subcortical structures, so far, the effect of its stimulation and/or inhibition on anesthesia has not been studied. Evidence for the altered state of connectivity of the claustrum during anesthesia was provided in a study by Smith et al. that was performed on rats under isoflurane anesthesia. They revealed a decreased connectivity of the claustrum with the cortex and thalamus when compared with the connectivity during the awake state [10].

In this paper, we have shifted our attention from the classical view of anesthetics working on different nervous system structures towards the view of the claustrum as a “conductor” of these structures [4]. In order to investigate the potential role of the claustrum in anesthesia, we evaluated the effect of claustral stimulation on depth of anesthesia in isoflurane-anesthetized rats.

## 2. Materials and Methods

This study was carried out in accordance with the recommendations of the European Communities Council Directive 86/609/EEC on the protection of animals used for scientific purposes. The protocol was approved by the French Ethical Committees, Ministry of National Education, Higher Education and Research, France (IBENS # c75-0520).

In this study, we used adult male Wistar rats (*N* = 5) with body weights between 250 and 300 g. The animals were housed under standard conditions with water and food ad libitum at a 12 h light–dark cycle.

### 2.1. Anesthetic Protocol for Surgery

Anesthesia was induced in the animal induction chamber (Classical T3 Vaporizer, SurgiVet, USA) using 2.5% isoflurane (Abbott Laboratory, US), with a delivery rate of 0.8 l/min for 5 min. The rats were placed in the stereotaxic instrument (Kopf: model 942, Kopf, Tujunga, CA, USA) and coupled to the facemask of the anesthesia apparatus in an open circuit. The anesthesia was thereafter maintained using 1.7% ± 0.21% isoflurane in air (FiO2 = 0.21), with spontaneous respiration.

### 2.2. Surgical Technique and Electrode Placement

The hair of the cranial region was removed, and the scalp was infiltrated with 0.2 mL 0.5% bupivacaine. After 5 min, a median line incision of 1 cm was performed, and the scalp was carefully dissected and removed. Bipolar electrocautery was used to maintain hemostasis.

A hole was stereotactically drilled into the skull at 1.76 mm anterior and 4 mm left lateral of bregma, corresponding to the coordinates of the claustrum [11]. A bipolar tungsten electrode with a distance between the two ends of the stimulating electrode of 0.5 mm, designed as such in order to obtain a more localized stimulation, was then carefully placed at a depth of 5.5 mm from the cortical surface, corresponding to the position of the claustrum. A ground electrode was placed on the left hind paw.

For electrocorticogram (ECoG) signal acquisition, three epidural silver electrodes were placed over the left hemisphere within holes drilled into the skull, as follows: two recording electrodes in the frontal cortex (4 mm anterior and 3 mm lateral of bregma) and parietal cortex (4 mm posterior and 3 mm lateral of bregma), and one reference electrode in the olfactory cortex (7 mm anterior and 2 mm lateral of bregma). Acquisition of the ECoG unfiltered signal was performed using a Tucker Davis Technology System 3 at a sample rate of 10 kHz.

### 2.3. The Stimulation of Claustrum under Slow-Wave Anesthesia

Isoflurane anesthesia was maintained in a slow-wave state before the stimulation started. The average isoflurane concentration necessary to maintain a similar depth slow-wave anesthesia for all rats was 1.7% ± 0.21%, and it was maintained constantly in each rat during stimulation and poststimulation.

The claustrum area stimulation was initiated after reaching a stabilized slow-wave state that lasted at least 10 min under a constant isoflurane concentration, specific to each rat. During the experiment, the anesthetic depth was assessed by visual inspection of the raw ECoG, and the presence of a continuous, unchanged slow-wave pattern for at least 10 min was considered the criterion for a constant anesthetic depth. Offline analysis of the data was also carried out using ECoG derived anesthetic depth parameters: median frequency (MEF), spectral edge frequency (SEF), and approximate entropy (AppEn). Twenty rectangular stimuli of 800 µA intensity and 1 s duration each were applied at interstimulus intervals of 10 s in one single recording session. In order to evaluate the electrical response of claustrum’s surrounding regions to the same stimulation protocol, the electrode was carefully repositioned (100 µm/s) 3 mm upward (at 5.2 mm from the cortical surface) and downward (at 5.8 mm from the cortical surface). In these regions, no burst suppression (BS) pattern was observed on the ECoG during the entire stimulation period. A stabilization period of 10 min between these stimulation sessions was maintained.

### 2.4. ECoG Data Analysis

The ECoG recordings were exported as *.mat files and were also converted and saved as *.acq files. We determined the time from the initiation of claustrum stimulation to deepening anesthesia (documented by the burst suppression (BS) pattern appearance), the duration of the profound anesthesia, and the burst count during the BS period. To assess BS anesthesia depth, we performed a burst count using custom routines implemented in Matlab™ (Mathworks Inc., Natick, MA, USA) by dividing the entire recording into segments of 10 s and calculating the average signal for each of these segments. Subsequently, the average signals were combined to obtain the final recording. The stages (slow-wave vs. BS) and depth of anesthesia were identified by visually inspecting ECoG signal spectrograms [12].

For the estimation of anesthesia depth in the non-BS state, we performed the calculation of ECoG derived parameters that are also used for anesthesia monitoring in clinical settings [13]—MEF (the EEG frequency of the power spectrum at which lower and higher frequencies are each 50% of the power) and SEF 90% (the EEG frequencies that account for 90% of the power spectra area). Additionally, we incorporated into the ECoG analysis a newly developed algorithm—AppEn (a measure of system complexity applied to EEG, known to correlate with changes in consciousness state: awake or asleep), which was verified in animal and human studies [14].

Data were segmented into prestimulation, pre-BS, and post-BS periods. ECoG analysis was performed on 8-second selected segments and included the spectral analysis derived parameters mentioned above. The AppEn was computed on six ECoG 8 s segments in each rat, with two before stimulation, two before BS onset, and two after BS end. The ECoG was resampled to 150 Hz and a 1–40 Hz bandpass filter was applied. Each ECoG sample consisted of 900 consecutive data points (8 s) free from stimulation artifacts. The pre-BS and post-BS ECoG samples were chosen in the middle (8 s) of an artifact-free, slow-wave signal between two adjacent stimulations. The filtration factor r was set to 0.2 times the standard deviation of prestimulation ECoG. The length of the signal sequences compared for AppEn estimation was set to 3 (*m* = 3).

The AppEn was estimated using a custom implementation in Matlab (Mathworks, Inc., Natick, MA, USA) of the algorithm described by Pincus et al. [15]. MEF and SEF were computed using AcqKnowledge 4.2 software on 6 s length ECoG using epochs of 2-second.

### 2.5. Histological Analysis

To histologically document the stereotactic placement of the stimulating electrode within the claustrum, the electrode was initially covered with a dye (Vybrant Dil Cell-Label, Thermo Fisher Scientific). At the completion of ECoG recordings, the anesthetized animals were given an overdose of pentobarbital (100 mg/kg, i.p.), then their brains were removed from the skull and preserved in a 4% formaldehyde solution in 10% sucrose for 3 days at 4 °C. Subsequently, successive coronal brain sections of 100 μm thickness were obtained using a microtome (Leica TM), and observed on a Leica microscope with 4x lenses. Photographs were captured using an integrated photo camera.

### 2.6. Statistical Analyses

For data analysis, we used SPSS 18.0 (IBM SPSS Statistics 21; IBM Corporation, Armonk, NY, USA). Data were presented as mean value ± standard deviation. For comparing MEF, SEF, and AppEn mean values during the prestimulation, pre-BS, and post-BS periods we used a one-way ANOVA test with Dunnet’s T3 post hoc test. The statistical significance threshold was set to *p* < 0.05.

## 3. Results

**Figure 1:**
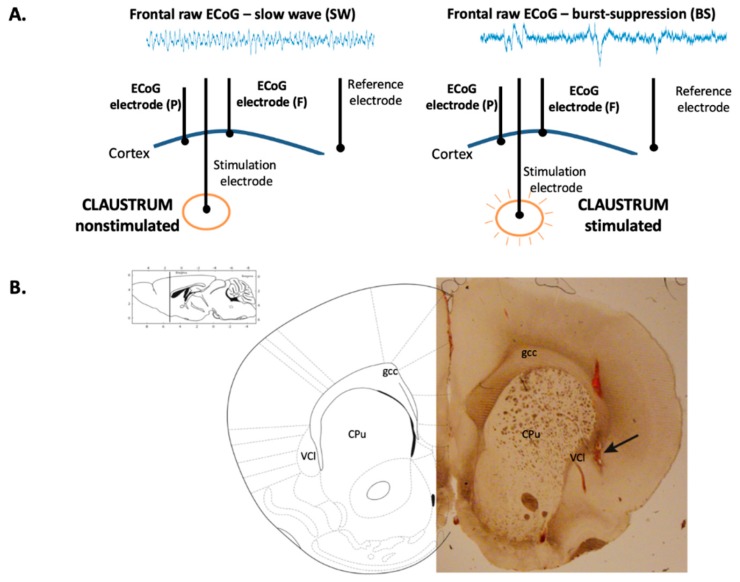
Electrodes placement and histological assessment. (**A**) Electrocorticogram (ECoG) features during nonstimulation and stimulation of claustrum. The placement of the electrodes for stimulation and recording in the frontal (F) and parietal (P) cortex are shown. (**B**) Histological image of a coronal rat brain slice presenting the stimulation electrode trace in claustrum (black arrow). Ventral claustrum (VCl), caudate putamen (CPu), genu of corp. call (gcc). (adapted from Paxinos and Watson (2009). The rat brain in stereotaxic coordinates. Amsterdam: Elsevier/Academic).

**Figure 2:**
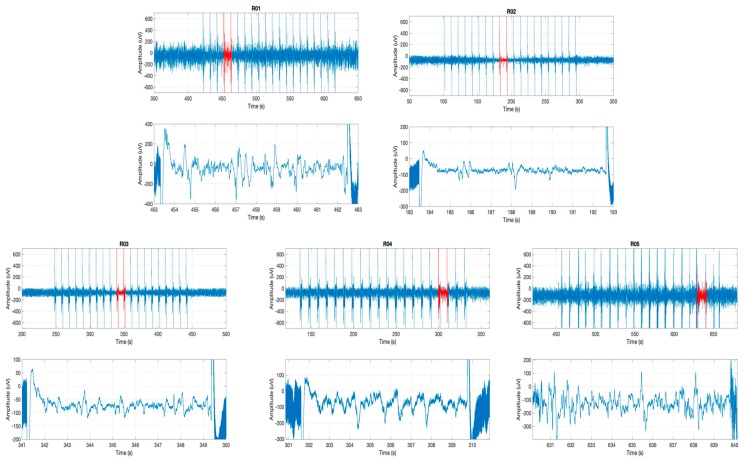
Electrocorticogram (ECoG) signals during stimulation from all five rats. Red landmark represents burst suppression (BS) period, which is presented as 10-second recordings below each ECoG. ECoG recordings are numbered from R01 to R05, corresponding to the rats included in this study.

**Figure 3:**
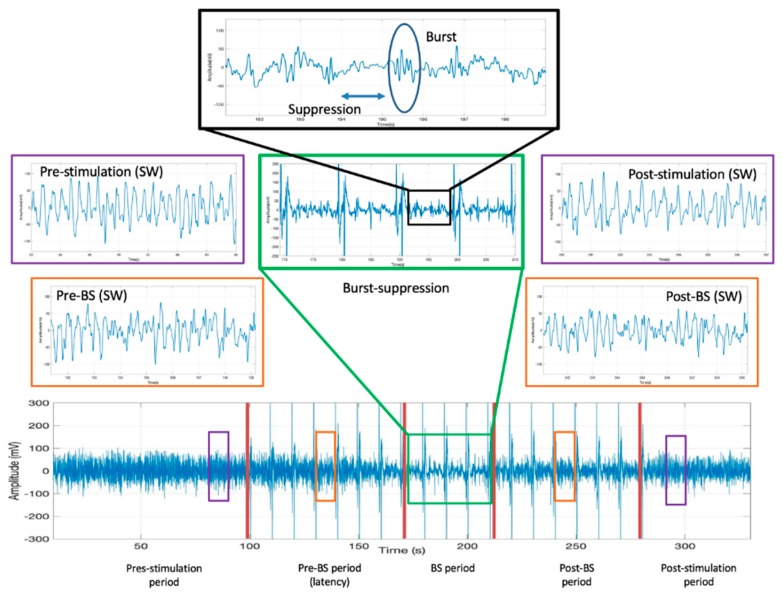
The full recording of a frontal left electrocorticogram from rat R02 with demarcation of the prestimulation, stimulation (with pre-BS, BS, and post-BS phases), and poststimulation periods. Vertical spikes presented over the electrocorticogram raw are electrical stimuli artifacts. The BS pattern is visible in the green and black boxes (showing the burst and suppression features). Slow-wave anesthesia during pre-BS and post-BS is seen in the orange boxes, and the prestimulation and poststimulation periods are presented in the violet boxes. BS—burst suppression.

Stimulation of the claustrum during slow-wave anesthesia produced a deepening of anesthesia characterized by the appearance of the BS pattern on the ECoG (Figure 1A), corresponding to a concentration of isoflurane of 2% ± 0.17%. The correct placement of the stimulation electrode within the claustrum was confirmed by histological assessment (Figure 1B). The ECoG recordings showing the BS pattern for each rat investigated are presented in Figure 2. The BS pattern started 57.6 ± 24.6 s after the beginning of claustrum stimulation and included a burst count of 11.6 ± 3.97 s and lasted for 37.2 ± 10.3 s (Figure 3).

**Figure 4:**
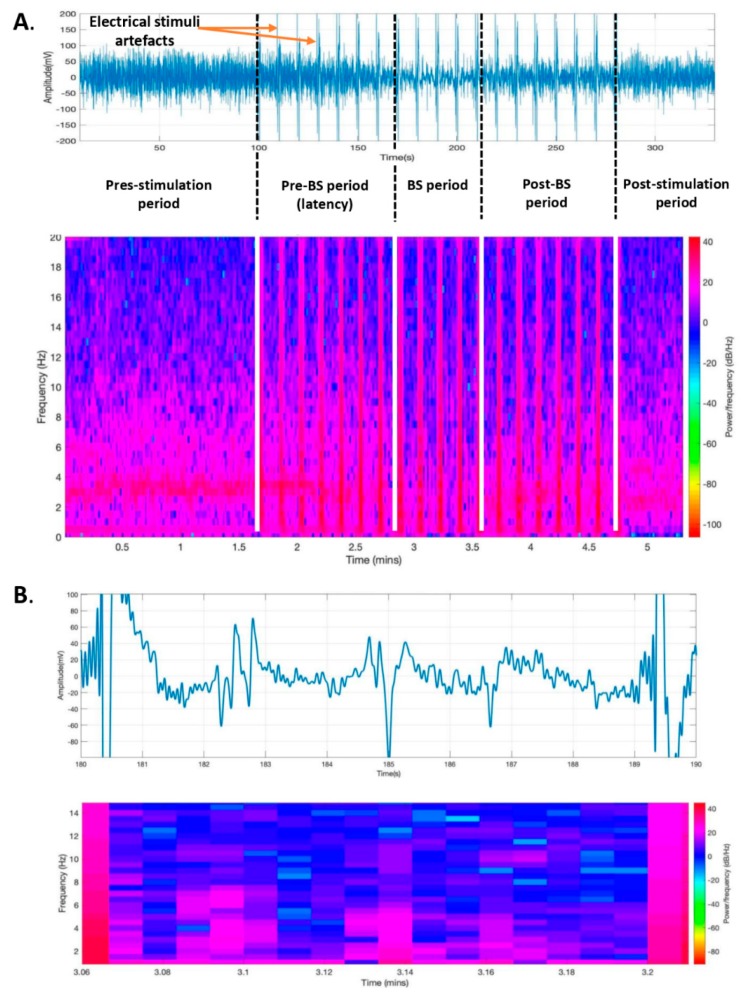
Electrocorticogram changes during claustrum stimulation. (**A**) Frontal left electrocorticogram with its corresponding spectrogram during the prestimulation, stimulation (with pre-BS, BS, and post-BS phases), and poststimulation periods. (**B**) A detailed view of the spectrogram on an 8-second electrocorticogram recording during the BS period between two electrical stimuli. BS—burst suppression.

Changes in the depth of the anesthesia during the entire recording were also described by spectrogram analysis (Figure 4A). A decrease in spectral power during the suppression period (blue color) and an increase in spectral power during bursts (purple color) were observed on the spectrogram on 8-second ECoG epochs during the BS period (Figure 4B).

**Figure 5:**
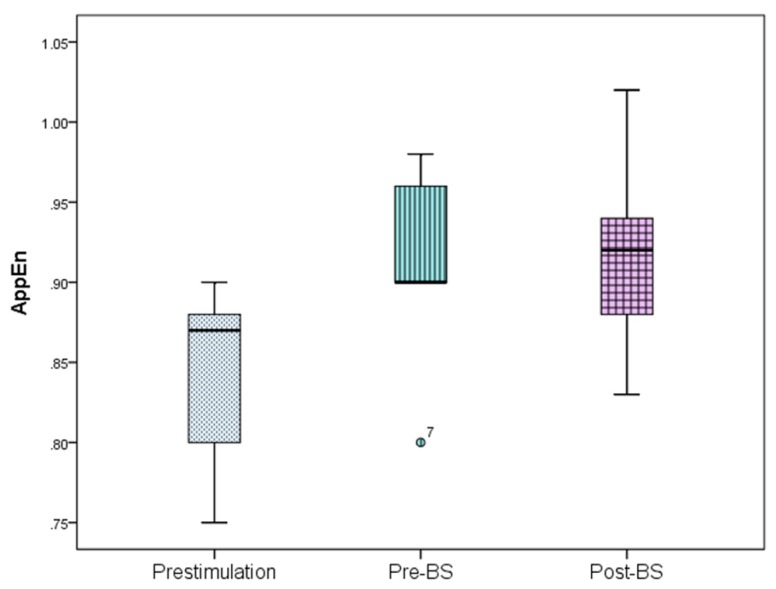
Approximate entropy (AppEn) variation for the prestimulation, pre-BS, and post-BS periods. Error bars represent standard deviation. One-way ANOVA analysis revealed no statistical significance between these periods (*p* > 0.05). BS—burst suppression.

**Figure 6:**
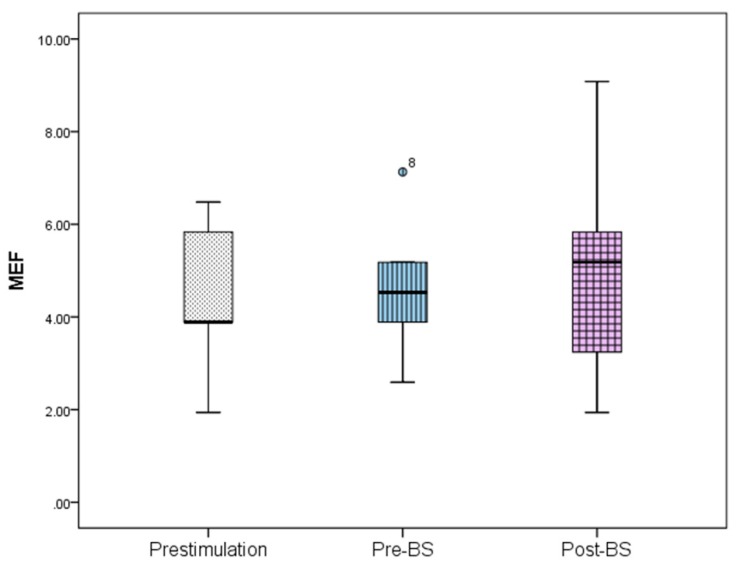
Median frequency (MEF) variation for the prestimulation, pre-BS, and post-BS periods. Error bars represent standard deviation. One-way ANOVA analysis revealed no statistical significance between these periods (*p* > 0.05). BS—burst suppression.

**Figure 7:**
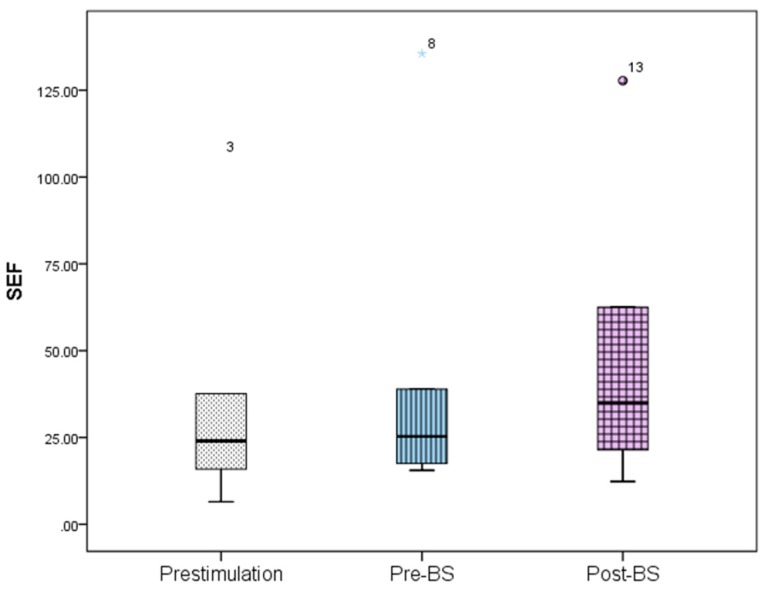
Spectral edge frequency (SEF) variation for the prestimulation, pre-BS period, and post-BS period. Error bars represent standard deviation. One-way ANOVA analysis revealed no statistical significance between these periods (*p* > 0.05). BS—burst suppression.

**Figure 8:**
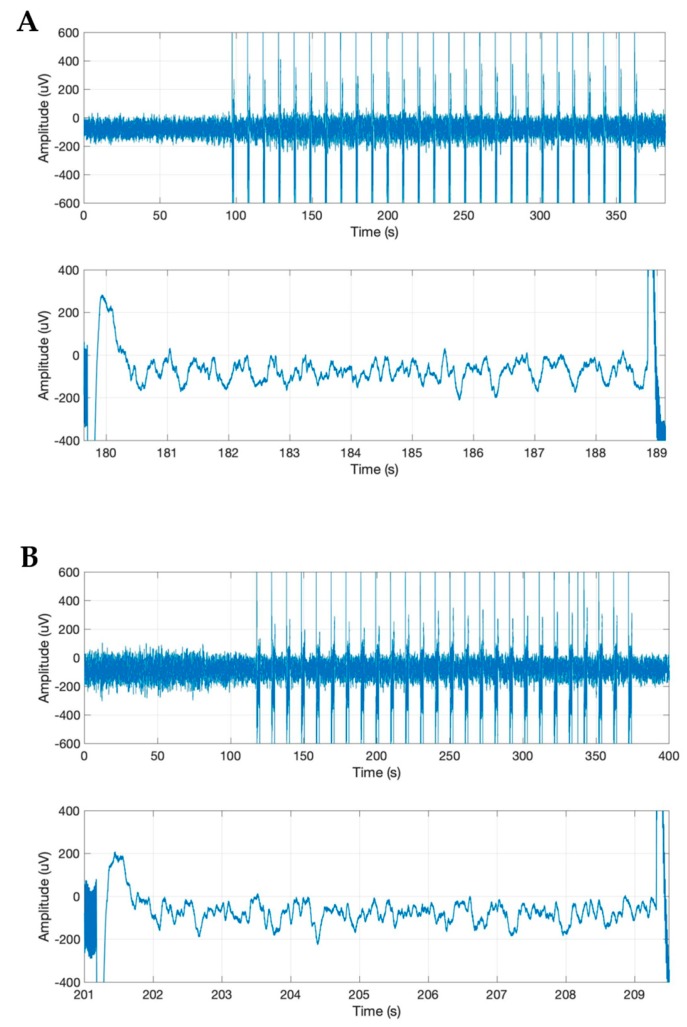
Frontal electrocorticogram (ECoG) features during stimulation of the claustrum surrounding areas showing slow-wave activity with no burst suppression pattern appearance. (**A**) ECoG slow-wave activity during stimulation of the area located at 5.2 mm below the cortex surface, 1.76 mm anteroposterior, and 4 mm medio-lateral. (**B**) ECoG slow-wave activity during stimulation of the area located at 5.8 mm below the cortex surface, 1.76 mm anteroposterior, and 4 mm mediolateral.

**Figure 9:**
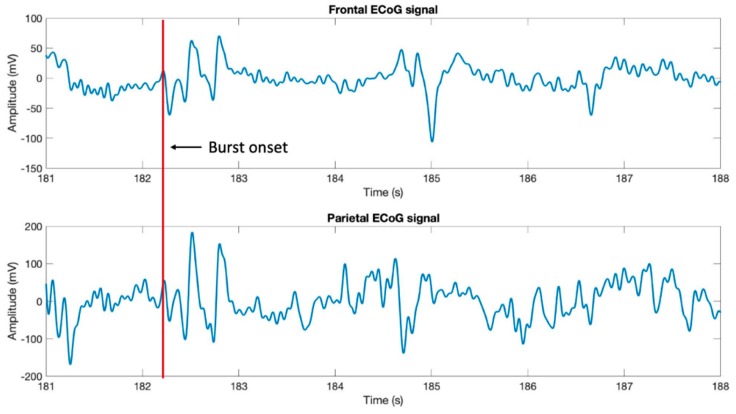
Left frontal and parietal electrocorticogram signals. These recordings show a simultaneous appearance in burst suppression pattern, as marked by the red line.

The ECoG changes in the slow-wave anesthetic depth prestimulation, pre-BS, and post-BS were analyzed using AppEn, MEF, and SEF, while data processing was done using one-way ANOVA. AppEn had a prestimulation value of 0.84 ± 0.06, a pre-BS value of 0.91 ± 0.07, and a post-BS value of 0.92 ± 0.07 (Figure 5). In the case of MEF, the values obtained were 4.41 ± 1.79 Hz prestimulation, 4.66 ± 1.67 Hz pre-BS period, and 5.05 ± 2.73 Hz post-BS period (Figure 6). For SEF, data yielded the following values: 37.93 ± 39.56 Hz prestimulation, 46.55 ± 50.57 Hz pre-BS, and 51.79 ± 46.49 Hz for the post-BS period (Figure 7). These results revealed a constant anesthetic depth during the prestimulation, pre-BS, and post-BS periods (*p* > 0.05). There was no burst suppression pattern during the entire stimulation period when the same 20 stimuli were applied above (5.2 mm) and below (5.8 mm) claustrum (Figure 8). Comparative analysis of the left frontal and parietal ECoG signals revealed the simultaneous appearance of the BS pattern (Figure 9).

## 4. Discussion

In cases of anesthesia, to our knowledge, there are no studies on the impact of stimulation of the claustrum on anesthetic state. Considering the acknowledged connection between the left anterior claustrum and the frontal–parietal cortex, known to be involved in maintaining the state of consciousness and anesthesia and its strong connection with cingulate cortices [16,17], we chose to stimulate the left anterior claustrum during anesthesia. We noticed that claustral stimulation during anesthesia leads to the appearance of the BS pattern, used as a sign of increased anesthetic depth. It is known that the effect of electrical stimulation on a neuron’s function is deepened by the electric field intensity; however, the exact mechanism of action is not thoroughly understood yet, regardless of the use of direct or alternating current. For instance, it was observed and reported that brain stimulation at 40 mA induces anesthesia (electroanesthesia) and stimulation at 3–10 mA induces a deep sleep state and possibly a suppression of epileptic activity in rats and humans [18,19,20,21]. Thus, we considered that sustained stimulation of the claustrum induced an inhibition. Further, the claustrum consists of numerous excitatory claustrocortical neurons and 10–15% inhibitory interneurons, and its activation is associated with increased cortical activity corresponding to rapid eye movement (REM) during sleep [22,23]. However, we cannot exclude the spread of electric stimuli to surrounding structures such as insular cortex. We suggest that a possible decrease in excitatory function of the claustrum, due to its electrical stimulation, reduces the anesthetic requirement, so that the BS pattern is observed at the same concentration of isoflurane as required for maintaining slow-wave anesthesia. The BS pattern observed on an ECoG in anesthesia represents a succession of bursts (high positive and negative amplitude changes) interpolated by isoelectric activity, a condition also observed in states of coma, hypothermia, brain hypoxia, and traumatic brain injury. The mechanisms underlying the BS phenomenon are still under investigation; however, two theories have been suggested. One theory from Amzica et al. [24] states that the BS pattern appears due to a change in calcium concentration in the brain, as the influx of calcium into neurons during intense depolarization causes the burst, while the calcium depletion in the extracellular space determines the appearance of the isoelectric activity. Another theory from Brown et al. [25] considers that BS is a metabolic phenomenon mediated by changes in ATP level. Thus, ATP depletion during bursts induces increased activity of KATP channels with secondary hyperpolarization of the neuronal membrane, which explains the isoelectric activity following bursts. During anesthesia, the BS pattern is observed during deep anesthetic stages (bispectral index < 21) and is quantified by burst suppression ratio (BSR)—the time of suppression for 63 s and burst count (BC)—the number of bursts per minute [26]. During surgical anesthesia, the patient is not usually maintained in BS.

Although our study cannot be directly related to Koubeissi et al. [5], due to their different type and duration of stimuli and the awake state of the patient, we consider that our findings add new insights into understanding the function of the claustrum. The BS pattern was transitory, lasting about 30 s, unlike in the Koubeissi study, which showed a permanent loss of consciousness during the claustral stimulation, in a patient who was awake, not anesthetized. The transient change in ECoG in our study may be due to the different type of electrical stimuli, compared to those applied by Koubeissi et al. [5] (biphasic waves at 14 mA, 50 Hz, and 0.2 ms pulse width). Furthermore, we only stimulated the left claustrum and its function may have been taken over by the right one. On the other hand, they stimulated the claustrum continuously for a maximum of 10 s, while we stimulated it intermittently for 200 s. Another difference is the presence of a delay of about 57.6 ± 24.6 s in the BS pattern appearance from the onset of electrical stimulation in our study, compared with that of Koubeissi et al. [5], where the loss of consciousness occurred immediately after electrical stimulation. The delay in BS onset could be associated with the time required for claustrum to reach an inhibited state following electrical stimulation.

Nevertheless, Bickel and Parvizi observed in their study performed in humans (*N* = 5) that electrical stimulation of the posterior part of the claustrum induced sensory-motor effects but failed to induce a loss of consciousness [6]. We consider that differences between the results of the two studies stem primarily from different protocol choices since Bickel and Parvizi opted for a rather heterogeneous protocol compared to Koubeissi et al. [5], specifically tailoring the stimulation to the needs of each of their five participants. Therefore, in the study showing no loss of consciousness, the stimulus intensity varied from 2 mA to a maximum of 10 mA, while the length was kept the same, 1 s, for all subjects. On the other hand, in the study that concluded the existence of consciousness alterations, the intensity of all stimuli was 14 mA and the length ranged from 3 to 10 s. Moreover, there is evidence that high frequency stimulation of rat claustrum induced a decrease in attentiveness and reduced the ability to perform an operant task [27].

On the other hand, Hudetz et al. considered that the main role in anesthesia was played by the posterior cingulate cortex (thought to act as a hub), which forms a network with the lateral parietal cortex, anterior cingulate nucleus, medial prefrontal cortex, and intralaminar thalamic nuclei [28]. The arguments that support a central role of the claustrum in anesthesia are relying on its strong connections, especially with the cingulate cortex (claustral projections to cingulate cortices are spread throughout the extent of claustrum) and the somatosensory cortex, but also with the medial prefrontal cortex [16,29,30]. Recently, it was reported that the claustrum could be involved in the process that increases connectivity in the gamma frequency range between different cortical structures. Such information reinforces the idea that claustrum has its role in anesthesia, as the loss of consciousness during anesthesia associates with a decrease of connectivity in the gamma frequency range between the frontal and parietal cortex [31,32]. Moreover, a recent study of Smith et al. [10] revealed that during isoflurane anesthesia, functional connectivity between the claustrum, medial prefrontal cortex, and mediodorsal thalamus is decreased, the medial prefrontal cortex being referred to also in the paper of Hudetz et al. [28]. It is known that some nonvolatile anesthetics can interact with specific receptors. Ketamine acts on N-Methyl-D-aspartic acid (NMDA) receptors, opioids are agonist for µ and k receptors, and propofol, etomidate, and benzodiazepines potentiate activation of gamma-aminobutyric acid (GABA) receptors [33,34]. k-opioid, NMDA, dopaminergic and GABA receptors have been shown to be present at the level of claustrum [35,36,37]. Therefore, these receptors in claustrum could be anesthetic targets, with potential involvement in the claustrum response to anesthesia.

## 5. Conclusions

In conclusion, our results show that electrical stimulation in the area of the left anterior claustrum induces a transitory deepening of anesthesia with BS pattern occurrence during slow-wave isoflurane anesthesia in rats. Another feature observed is the presence of a delay between the beginning of the stimulation and BS pattern appearance. Although our results plead for a possible role of claustrum in anesthesia, further studies are required to elucidate the mechanisms involved.
